# Vaginal *Lactobacillus* Impair *Candida* Dimorphic Switching and Biofilm Formation

**DOI:** 10.3390/microorganisms10102091

**Published:** 2022-10-21

**Authors:** Carola Parolin, Vanessa Croatti, Barbara Giordani, Beatrice Vitali

**Affiliations:** Department of Pharmacy and Biotechnology, Alma Mater Studiorum-University of Bologna, 40127 Bologna, Italy

**Keywords:** *Lactobacillus*, *Candida*, biofilm, virulence factors, vaginal microbiota, vulvovaginal candidiasis

## Abstract

*Lactobacillus* spp. generally dominate the vaginal microbiota and prevent pathogen adhesion and overgrowth, including *Candida* spp., by various mechanisms. Although *Candida* spp. can be commensal, in certain conditions they can become pathogenic, causing vulvovaginal candidiasis. The insurgence of candidiasis is related to the expression of *Candida* virulence factors, including morphologic switching and biofilm formation. Germ tubes, pseudohyphae, and hyphae promote *Candida* tissue invasion, biofilms increase persistence and are often resistant to antifungals and host immune response. Here, we explored the inhibitory activity of vaginal *Lactobacillus* strains belonging to *Lactobacillus crispatus*, *Lactobacillus gasseri*, *Limosilactobacillus vaginalis*, and *Lactiplantibacillus plantarum* species towards *Candida* virulence factors. With the aim to investigate the interrelation between mode of growth and functionality, supernatants were collected from lactobacilli planktonic cultures and, for the first time, from adherent ones, and were evaluated towards *Candida* dimorphic switching and biofilm. *Candida* biofilms were analyzed by multiple methodologies, i.e., crystal violet staining, MTT assay, and confocal microscopy. *Lactobacillus* supernatants reduce *Candida* switching and biofilm formation. Importantly, *L. crispatus* supernatants showed the best profile of virulence suppression, especially when grown in adherence. These results highlight the role of such species as a hallmark of vaginal eubiosis and prompt its employment in new probiotics for women’s health.

## 1. Introduction

Among microorganisms colonizing the human vaginal cavity, *Candida* spp. are frequently retrieved. This yeast can also colonize the gastrointestinal tract, oral cavity, and skin, coexisting with other members of the local microbiota most of the time. However, owing to perturbations of the microbiota and changes in an individual healthy immune system or alteration of the local environment, *Candida* spp. can increase in cell load and cause infection [1]. At the vaginal level, *Candida* overgrowth and pathogenic feature acquisition cause vulvovaginal candidiasis (VVC), an infection characterized by itching or irritation of the vulva, pain, and abnormal vaginal discharge [2]. VVC affects 75% of the world’s female population at least once in their lifetime, and about 5–8% of these women suffer from recurrent VVC (RVVC) [3,4,5]. Most VVC cases (75–90%) are attributable to *Candida albicans*, but recently *Candida* non-*albicans* species (NAC) infections have been increasingly diagnosticated [2,6]. Similar to other pathogens, the clinical impact of *Candida* spp. could be related to their ability to grow in an adherent form, i.e., a biofilm, a dense community of cells immersed in a secreted matrix [3,7], although most studies involving vaginal *Candida* isolates are carried out in vitro [8,9,10]. Frequently, *Candida* biofilms are intrinsically resistant to conventional antifungal therapeutics and to host immune response, making biofilm-based infections a significant clinical challenge [1]. The evolution of vaginal *Candida* infections is also related to the dimorphism of this yeast, which consists in the morphologic switching of fungal round or ovoidal cells into elongated pseudohyphae and branched hyphal structures [11]. The capability of *Candida* spp. to undergo dimorphic switching and the ability to form complex biofilms are considered virulence factors, since pseudohyphal and differentiated hyphal forms, together with adherence to a surface, make *Candida* more invasive into human mucosae and resistant to antifungal treatments [1].

The vaginal microbiota is composed of aerobic and anaerobic microorganisms colonizing the epithelium. The resulting microbial diversity and multi-microbial interactions contribute to the maintenance of vaginal homeostasis [12,13]. In particular, *Lactobacillus* and related genera are dominant and abundant in the vaginal microbiota of healthy women. The probiotic and antimicrobial properties of individual species are well recognized and include the ability to prevent pathogen overgrowth, such as *Candida* spp. [14]. Indeed, it has been well-documented that lactobacilli can prevent or reduce *Candida* growth through multiple mechanisms. Among these, lactobacilli are reported to compete with pathogens for nutrients and adhesion sites on the host epithelium, as well as to release active compounds such as bacteriocins, hydrogen peroxide, and organic acids. In particular, the production of lactic acid is related to vaginal pH lowering, which impairs pathogen growth [15,16,17,18,19]. Many studies have dealt with the ability of vaginal lactobacilli to reduce *Candida* spp. growth in vitro [15,20], but enabling impairment of dimorphic switching and biofilm formation has been poorly investigated till now.

In the present paper, a collection of *Lactobacillus* strains of vaginal origin (belonging to *Lactobacillus crispatus*, *Lactobacillus gasseri*, *Limosilactobacillus vaginalis*, and *Lactiplantibacillus plantarum* species) was tested for the potential to reduce germ tube/pseudohyphal/hyphal development and biofilm formation of *Candida* spp. Notably, cell-free culture supernatants (CFS) collected from lactobacilli grown in planktonic (pk-CFS) and adherent form (bf-CFS) were employed to challenge *Candida* clinical isolates, in order to investigate the interrelation between mode of growth (planktonic/biofilm) and functionality. Both *Candida albicans* and NAC clinical isolates (*Candida glabrata*, *Candida lusitaniae*, *Candida tropicalis*, *Candida krusei*, and *Candida parapsilosis*) were employed to reflect the most common VVC etiological agents.

## 2. Materials and Methods

### 2.1. Lactobacilli Culture Conditions and Preparation of Planktonic/Biofilm Cell-Free Supernatants

*Lactobacilli* were isolated from vaginal swabs of healthy premenopausal Caucasian women, following the protocol approved by the Ethics Committee of the University of Bologna, Bologna, Italy (52/2014/U/Tess) [15]. Strains were identified and re-classified as *Lactobacillus crispatus* (BC1–BC8), *Lactobacillus gasseri* (BC9–BC14), *Limosilactobacillus vaginalis* (BC16–BC17), and *Lactiplantibacillus plantarum* (BC18–BC19). They were routinely cultured in 10 mL de Man, Rogosa, and Sharpe (MRS) broth (Difco, Detroit, MI, USA) supplemented with 0.05% L-cysteine (Merck, Milan, Italy), at 37 °C, in anaerobic jars containing GasPak™ (GasPak™ EZ Anaerobe Container System, Becton, Dickinson and Co., Sparks, MD, USA). For each strain, two sequential 24 h cultures were set up, then a 10^7^ CFU/mL suspension was prepared. Such suspensions were inoculated both in 10 mL glass tubes to obtain the planktonic (pk) cultures, and in 6-well plates (4 mL per well) to obtain the biofilm (bf) cultures. All cultures were incubated for 72 h at 37 °C, in anaerobiosis, then culture supernatants were recovered by centrifugation (2750× *g*, 10 min) and filtered through a 0.22 μm membrane filter to obtain cell-free supernatants (CFS, pk-CFS, and bf-CFS) [20].

### 2.2. Candida spp. Culture Conditions and Treatment with Lactobacilli Supernatants

*Candida* strains were isolated from vaginal swabs of premenopausal, VVC-affected women during routine diagnostic procedures at the ‘Microbiology Laboratory’ in Sant’Orsola-Malpighi University Hospital of Bologna, Bologna, Italy and belong to the Microbiology Laboratory yeasts collection [16,21]. *Candida* strains were identified as *Candida albicans* (SO1, SO2), *Candida glabrata* (SO17, SO18), *Candida lusitaniae* (SO22), *Candida tropicalis* (SO24), *Candida krusei* (SO26), and *Candida parapsilosis* (SO27) and were routinely cultured on Sabouraud Dextrose (SD, Difco) agar plates at 35 °C in aerobiosis. Following the protocols suggested by EUCAST guidelines [22], fungal suspensions were prepared in sterile water at an absorbance (measured at 600 nm) of 0.8–1.3, then diluted 1:10 in RPMI 1640 medium (Euroclone, Milan, Italy) buffered to pH 7.0 with 0.165 M MOPS (morpholinepropanesulfonic acid buffer, Merck), and added with 2% glucose (Merck) to obtain 1–5 × 10^6^ *Candida* CFU/mL. Such suspensions were inoculated in flat-bottomed 96-well plates (0.1 mL per well), added with the same volume of each *Lactobacillus* pk-CFS and bf-CFS. Growth control wells were set up by adding the same amount of MRS medium to *Candida* suspensions. Plates were incubated at 35 °C in aerobic conditions. Each condition was tested at least in triplicate.

### 2.3. Evaluation of Candida Dimorphic Switching

*Candida* suspensions treated with *Lactobacillus* pk-CFS and bf-CFS were evaluated after 48 h of culture for dimorphic switching by observing a sample aliquot under a light microscope using a 20× objective (Eclipse 90i microscope, Nikon Instruments, Europe BV, Amsterdam, The Netherlands). At least three microscopic fields per sample were observed for the presence of round and elongated yeast cells (i.e., germ tubes, pseudohyphae, and hyphae) and samples treated with CFS were compared to untreated samples (growth control). *Candida* growth controls showed the occurrence of dimorphic switching except for *C. glabrata* SO17 and SO18, which were excluded from this analysis. If CFS treatment determined the disappearance of elongated yeast cells in at least two microscopic fields, the treatment was considered as inhibitory of dimorphic switching. On the contrary, in the presence of elongated yeast cells in at least two microscopic fields, CFS was considered as non-inhibitory.

### 2.4. Evaluation of Candida Biofilms

Here, 96-well plates inoculated with *Candida* suspensions and *Lactobacillus* pk-CFS and bf-CFS were incubated at 35 °C for 72 h to allow biofilm formation. At the end, floating cells were removed and adherent biofilms were gently washed with PBS twice. Three replicated 96-well plates were prepared. The first plate was used for biofilm biomass quantification by crystal violet staining, the second plate was used for biofilm metabolic activity quantification by MTT assay, and the third plate for biofilm visualization by confocal microscopy, as described below.

#### 2.4.1. *Candida* Biofilm Quantification by Crystal Violet Staining

Biofilms were quantified by crystal violet staining as previously described, with minor modification [18]. Briefly, adherent cells were fixed with 200 μL of 99% ethanol (Merck) for 15 min then stained for 5 min with 100 μL of 1% (*w*/*v*) crystal violet (Merck) in 12% ethanol. Wells were washed with PBS three times to rinse out excess staining, then plates were air-dried. Crystal violet bound to the adherent yeast cells was resolubilized in 200 μL of 33% (*v*/*v*) ethanol and the absorbance was measured at 595 nm using a GENios Plate Reader (Tecan Group Ltd., Männedorf, Switzerland). Biofilm formation inhibition was calculated as follows:

%inhibition = [1 − (Abs T/Abs C)] × 100(1)

where Abs T represents the absorbance of the well subjected to a treatment with CFS and Abs C is the absorbance of growth control wells.

#### 2.4.2. *Candida* Biofilm Quantification by MTT Assay

*Candida* biofilms were incubated with 0.5% 3-(4,5-dimethyl-2-thiazolyl)-2,5-diphenyl-2H-tetrazolium bromide (MTT, 100 μL/well) diluted in culture medium at 35 °C for 30 min–4 h, depending on *Candida* species. Incubation time was optimized as 4 h for *C. albicans*, 3 h for *C. glabrata,* 30 min for *C. lusitaniae* and *C. parapsilosis*, and 2 h for *C. tropicalis* and *C. krusei*. For time optimization, see Supplementary Figure S1. At the end of the incubation time, supernatants were removed from wells and replaced by the same amount of isopropanol to solubilize the formed formazan salt. The absorbance was measured at 570 nm using a GENios Plate Reader. Biofilm formation inhibition was calculated following Equation (1) [23].

#### 2.4.3. *Candida* Biofilm Visualization by Confocal Microscopy

*Candida* spp. biofilms were stained using the Yeast Live-or-Dye Fixable Live/Dead staining kit, according to the manufacturer’s instructions (Biotium, Inc., Fremont, CA, USA). Briefly, samples were incubated with diluted dyes for 30 min at room temperature, then biofilms were air-dried for 15 min. Biofilms were visualized using a Nikon A1+ point confocal laser scanning microscope (CSLM) integrated with NIS-Elements C Software (Nikon Instruments), equipped with a Plan Apo λ 20× objective directly on the plastic support of the 96-well plate (λ exc = 489.3 nm and 561.4 nm).

### 2.5. Data Analysis

Experiments were repeated at least twice, and each condition was tested at least in triplicate. Data were analyzed by using GraphPad Prism version 9.2.0 for Windows (GraphPad Software, San Diego, CA, USA). Biofilm inhibition data were compared by ANOVA for multiple comparisons and Wilcoxon paired-rank test. A *p*-value < 0.05 was considered significant. Average values ± SEM are reported in the text and figures.

## 3. Results

### 3.1. Lactobacilli CFS Impair Candida Dimorphic Switching

**Figure 1:**
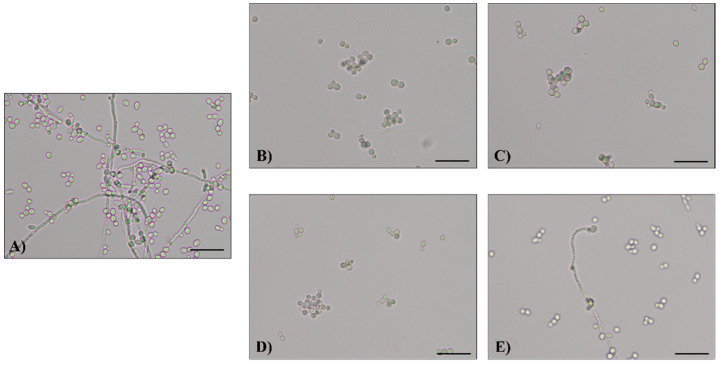
Representative images of *C. albicans* SO1 cell morphology obtained by optical microscope (20×): (**A**) growth control, (**B**) sample treated with pk-CFS from *L. crispatus* BC3, (**C**) sample treated with bf-CFS from *L. crispatus* BC3, (**D**) sample treated with pk-CFS from *L. gasseri* BC9, (**E**) sample treated with bf-CFS from *L. gasseri* BC9. Scale bar = 50 μm.

Culture supernatants collected from vaginal lactobacilli grown in planktonic (pk-CFS) and biofilm (bf-CFS) forms were tested for their capability to impair the dimorphic switch of *Candida* spp. Germ tubes, pseudohyphae, and hyphae formation or inhibition were qualitatively evaluated by microscopic observation of *Candida* cultures, and the results are summarized in Table 1. Some representative micrographs are reported in Figure 1 and Supplementary Figure S2. *C. albicans* isolates SO1 and SO2 developed germ tubes, pseudohyphae, and hyphae when grown in control conditions (i.e., *Candida* suspension in supplemented RPMI added with MRS medium). The treatment of *C. albicans* SO1 with lactobacilli CFS caused the inhibition of germ tube, pseudohyphae, and hyphae formation for most of the CFS analyzed, with the only exception being bf-CFS from *L. gasseri* BC9, pk- and bf-CFS from *L. vaginalis* BC16, and BC17 and pk-CFS from *L. plantarum* BC19. Similarly, dimorphic switching of *C. albicans* SO2 was inhibited by most *Lactobacillus* CFS, except for pk-CFS from *L. crispatus* BC3 and BC8, bf-CFS from *L. gasseri* BC9, pk- and bf-CFS from *L. gasseri* BC13, and from *L. vaginalis* BC16. *C. lusitaniae* SO22 produced an elongated-shape cellular form that we defined as an early germ tube. For this clinical isolate, treatment with most CFS did not abolish this morphologic form, except for CFS mainly from *L. crispatus* strains, i.e., CFS from *L. crispatus* BC1, BC5, BC6, and bf-CFS from *L. crispatus* BC3, BC4, and BC8. As for *L. gasseri* species, only pk-CFS from BC9 and CFS from BC10 and BC13 were able to inhibit *C. lusitaniae* SO22 dimorphic switching. *C. tropicalis* SO24 and *C. krusei* SO26 control cultures showed germ tubes and hyphae coexisting with ovoidal yeast cells. *C. tropicalis* SO24 switching was not inhibited by any *Lactobacillus* CFS. When *C. krusei* SO26 was treated with CFS, especially with CFS derived from strains of *L. crispatus* (BC1, BC4, BC5, BC6, and BC7) and *L. plantarum* (BC10, bf-CFS from BC11, and pk-CFS from BC14), dimorphic switching inhibition was observed. The formation of germ tubes and pseudohyphae by *C. parapsilosis* SO27 was inhibited by bf-CFS (but not pk-CFS) from all tested *L. crispatus* and *L. plantarum* strains and by pk-CFS from *L. gasseri* BC9. *C. glabrata* SO17 and SO18 did not show dimorphic switching in our experimental conditions, and thus its inhibition by CFS was not evaluated.

To better analyze CFS effects on *Candida* spp. dimorphism, CFS were grouped for *Lactobacillus* species and mode of growth (pk/bf), and inhibition was expressed in percentage form, calculated as the number of inhibitory cases (corresponding to “+” in Table 1) over the total cases for each category (*Lactobacillus* species/mode of growth), considering all *Candida* isolates together. CFS recovered from *L. crispatus* strains were more effective than those from *L. gasseri*. In addition, for *L. crispatus* species, bf-CFS were active in 32 out of 42 cases (corresponding to 76%) and pk-CFS were able to inhibit *Candida* spp. dimorphic switching in 20 out of 42 cases (48%). On the other hand, CFS from *L. gasseri* strains looked more active when recovered from planktonic cultures than the biofilm counterparts, since 17 pk-CFS out of 36 (47%) and only 13 bf-CFS out of 36 (36%) inhibited *Candida* dimorphic switching. A few CFS from *L. vaginalis* turned to inhibit dimorphic switching (2 out of 12, corresponding to 17%, for pk-CFS and 1 out of 12, corresponding to 8%, for bf-CFS) while for *L. plantarum* species pk-CFS were active in 5 out of 12 cases (42%) and bf-CFS in 7 out of 12 cases (58%), resembling *L. crispatus* CFS behavior.

### 3.2. Lactobacilli CFS Impact on Candida Biofilms

The effect of lactobacilli CFS on *Candida* spp. biofilm formation was examined by different methodologies. Indeed, *Candida* biofilms were quantified by two different methods, namely, crystal violet staining and MTT assay, that provide different and complementary information on the fungal biofilms. Crystal violet is a non-specific dye which binds cellular components as well as the extracellular matrix, allowing quantification of the total biofilm structure, including live cells, dead cells, and their secreted material. MTT is enzymatically reduced and converted into a formazan salt, allowing the selective quantification of metabolically active fungal cells.

**Figure 2:**
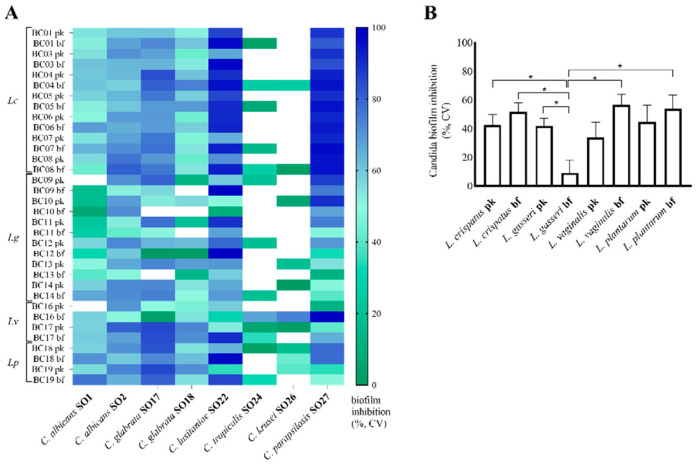
Inhibition of *Candida* isolate biofilm formation by lactobacilli pk-CFS and bf-CFS, evaluated by crystal violet (CV) staining. (**A**) Heatmap of biofilm inhibition percentage, calculated over corresponding *Candida* growth control. White squares indicate the absence of inhibition and stimulation of biofilm formation. (**B**) Average ± SEM of biofilm inhibition percentages, grouped for *Lactobacillus* species and mode of growth (pk/bf). Lc, *Lactobacillus crispatus*; Lg, *Lactobacillus gasseri*; Lv, *Limosilactobacillus* *vaginalis*; Lp, *Lactiplantibacillus* *plantarum*. * *p* < 0.05 (ANOVA).

Results obtained by crystal violet staining of *Candida* spp. biofilm subjected to different CFS treatments were collected and biofilm inhibition was calculated and reported in Figure 2A. Overall, *C. lusitaniae* SO22 and *C. parapsilosis* SO27 biofilms were the most susceptible to lactobacilli CFS, showing the highest inhibition percentages (inhibition average 74.8 ± 3.8% and 71.7 ± 4.3%, respectively). In particular, regarding *C. lusitaniae* SO22 biofilm, inhibition percentages over 95% were measured for bf-CFS from *L. crispatus* BC1, BC3, BC4, and *L. gasseri* BC9. bf-CFS from *L. crispatus* BC7, BC8, and *L. vaginalis* BC16 reached 95% inhibition percentages on *C. parapsilosis* SO27 biofilms. On the contrary, *C. tropicalis* SO24 and *C. krusei* SO26 biofilms showed the lowest inhibition profile. Indeed most *Lactobacillus* CFS (21 and 24 CFS out of 34, respectively) caused an increase in CV staining, suggesting a biofilm stimulating effect. Stimulation averages of 17.5 ± 7.6% and 28.5 ± 10.3% were calculated for *C. tropicalis* SO24 and *C. krusei* SO26, respectively. Intermediate profiles of inhibition were registered for *C. albicans* and *C. glabrata* isolates, corresponding to inhibition averages of 47.7 ± 3.5% and 65.4 ± 1.6% for *C. albicans* SO1 and SO2, and 62.0 ± 5.0% and 41.1 ± 6.7% for *C. glabrata* SO17 and SO18, respectively. In Figure 2B, biofilm inhibition values are grouped for lactobacilli species and pk/bf growth form and expressed as averages. Comparing CFS groups, bf-CFS recovered from *L. gasseri* strains proved to be significantly less effective in *Candida* biofilm inhibition than CFS from *L. crispatus* and bf-CFS from *L. vaginalis* and *L. plantarum* (*p* < 0.05, ANOVA, Figure 2B). In addition, comparing pk-CFS and bf-CFS recovered from the same *Lactobacillus* species, bf-CFS from *L. crispatus* and *L. gasseri* proved to be significantly more effective than the pk-CFS counterpart (*p* < 0.05, Wilcoxon paired-rank test).

**Figure 3:**
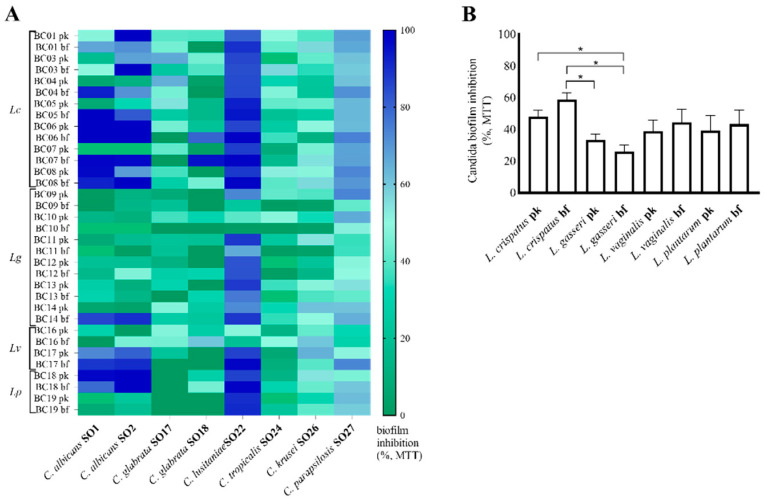
Inhibition of *Candida* isolate biofilm formation by lactobacilli pk-CFS and bf-CFS, evaluated by MTT assay. (**A**) Heatmap of biofilm inhibition percentage, calculated over corresponding *Candida* growth control, (**B**) average ± SEM of biofilm inhibition percentages, grouped for *Lactobacillus* species and mode of growth (pk/bf). Lc, *Lactobacillus crispatus*; Lg, *Lactobacillus gasseri*; Lv, *Limosilactobacillus* *vaginalis*; Lp, *Lactiplantibacillus* *plantarum*. * *p* < 0.05 (ANOVA).

Biofilm formation and CFS inhibition activity were also analyzed in terms of residual *Candida* spp. viability, quantified by MTT assay. Results are reported as a heatmap in Figure 3A. Overall, *C. lusitaniae* SO22 biofilms were the most susceptible to lactobacilli CFS (inhibition average 78.1 ± 4.1%), followed by *C. parapsilosis* SO27 (inhibition average 57.8 ± 2.2%), in accordance with data obtained by crystal violet staining. Additionally in this case, inhibition percentages over 95% were reached for bf-CFS but not for pk-CFS, namely, *L. crispatus* BC6, BC7, BC8, *L. vaginalis* BC17, and *L. plantarum* BC18 bf-CFS almost completely abolished *C. lusitaniae* SO22 biofilm (inhibition percentages 98.7–99.1%). *C. albicans* SO1 and SO2 showed intermediate susceptibility (inhibition average 49.3 ± 7.3% and 53.5 ± 6.8%, respectively). *C. tropicalis* SO24 and *C. krusei* SO26 biofilms were inhibited by *Lactobacillus* CFS (inhibition average 27.9 ± 3.1% and 38.2 ± 2.9%, respectively), suggesting that CFS were able to reduce *Candida* cell viability in the biofilm, although biofilm staining by CV highlighted biofilm stimulation. Figure 3B reports biofilm inhibition average grouped for lactobacilli species and pk/bf growth form, and the inhibition profile resembles that obtained with CV staining data. Considering MTT results, CFS from *L. crispatus* showed the highest biofilm inhibition averages, and also in this case bf-CFS appeared more active than pk-CFS, although this difference was not significant. CFS from *L. gasseri* strains reduced the *Candida* viable component of biofilm to a lesser extent, especially for bf-CFS that were significantly less active than CFS from *L. crispatus* (*p* < 0.05, ANOVA, Figure 3B). Intermediate inhibition activity was observed for *L. vaginalis* and *L. plantarum* CFS, with slightly higher activity for bf-CFS with respect to pk-CFS.

**Figure 4:**
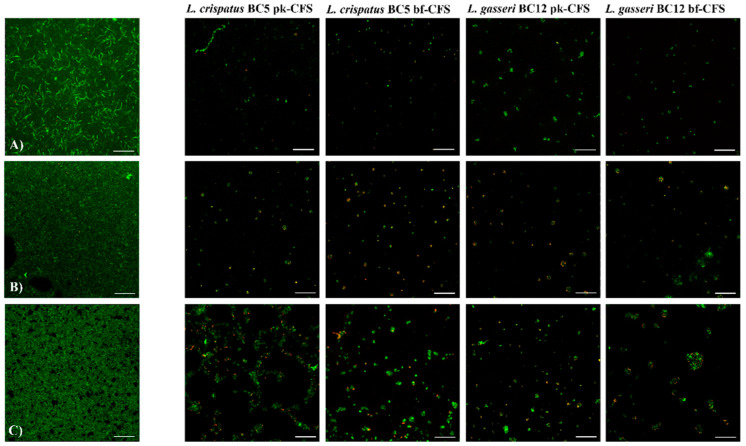
Confocal microscopy analysis of *Candida* biofilm treated with pk-CFS and bf-CFS from *L. crispatus* BC5 and *L. gasseri* BC12 (second—fifth columns). Biofilms were stained using two fluorescent dyes: thiazole orange (viable cells, green) and Live-or-Dye™ (dead cells, red). (**A**) *C. albicans* SO1 biofilm; (**B**) *C. lusitaniae* SO22 biofilm; (**C**) *C. tropicalis* SO24 biofilm. Scale bar = 100 μm.

*Candida* biofilms were also visualized by means of confocal microscopy, by using a dual fluorescent staining in order to discriminate between live and dead cells. Pk-CFS and bf-CFS recovered from *L. crispatus* BC5 and *L. gasseri* BC12 were chosen as model CFS to better investigate the effect of CFS from different *Lactobacillus* species. Figure 4 reports some representative confocal micrographs. *C. albicans* SO1 formed a thick biofilm, made up of complex aggregates of viable ovoidal cells and hyphae; the treatment with *Lactobacillus* CFS determined the biofilm disintegration. In particular, CFS from *L. crispatus* BC5 caused the complete loss of biofilm structure, and the staining highlighted few viable cells together with dead cells. The treatment with CFS from *L. gasseri* BC12 also impaired *Candida* biofilm formation, and only clusters of viable cells could still be observed. *C. lusitaniae* SO22 also built a continuous cellular mat with a dense extracellular matrix. The biofilm structure was completely dispersed by *Lactobacillus* CFS treatment, especially when *L. crispatus* BC5 CFS was used. Regarding *C. tropicalis* SO24, it formed a complex adherent biofilm, composed of viable cells immersed in a dense bright extracellular matrix. The treatment with *Lactobacillus* CFS caused the loss of biofilm architecture, although aggregates of adherent cells and extracellular matrix were still present. In particular, larger cell clusters were observed when *C. tropicalis* SO24 biofilm was treated with pk-CFS from *L. crispatus* or bf-CFS from *L. gasseri*. A similar pattern was observed for *C. krusei* SO26. Similar effects of biofilm inhibitory activity were observed for the other *Candida* isolates (Supplementary Figure S3).

## 4. Discussion

Vulvovaginal candidiasis and recurrent forms are highly prevalent infections in the female population, especially during reproductive age. Clinical symptoms associated with VVC negatively impact on women’s quality of life and ultimately turn into high social costs, in terms of request for medical assistance and lost productivity [24,25]. Treatment of VVC cases is generally based on azole antifungal drugs (mainly clotrimazole, miconazole, and fluconazole), topically or orally administered; up to 6 months’ azole maintenance therapy is recommended for RVVC cases (https://www.cdc.gov/std/treatment-guidelines.html (accessed on 1 July 2022)). However, antifungals generally are not able to eradicate vaginal pathogenic yeasts, but only exert cytostatic activity [26]. This is especially true when the infection is related to the establishment of a microbial biofilm, where pathogen cells are immersed in an extracellular matrix made up of complex polymers and thus partially or totally unattainable to antifungal drugs [27,28]. In addition, the morphologic switch of *Candida* cells to germ tube and hyphal forms represents a step of *Candida* spp. maturation towards a phenotype with increased invasiveness into tissues, and resistance to environmental and chemical stresses [7,29]. The formation of *Candida* biofilms has been related to the acquisition of resistance to antimicrobials, and adherence and morphology switching are considered important virulence factors [1,9]. With this in mind, a strategy effective towards *Candida* infections should be able not only to reduce *Candida* growth in the floating cellular form, but also to impair *Candida* biofilm formation and hyphae development. For these reasons, in vitro biofilm study is gaining importance as a platform to assess the potential of an active compound.

In the present paper, CFS already characterized for their antagonistic effect towards *Candida* planktonic growth were further analyzed for their anti-biofilm and anti-switching potential. In particular, CFS were recovered from vaginal *Lactobacillus* strains and tested against VVC *Candida* isolates; moreover, both lactobacilli pk-CFS and bf-CFS were employed, since the mode of growth of beneficial microbes themselves influences CFS functionality, as recently demonstrated [20,30]. To the best of our knowledge, spent supernatants recovered from adherent cultures of lactobacilli have never been tested towards *Candida* virulence factors, and this issue represents an important novelty of the present study. It is worth pointing out that the predominant microbial mode of growth in nature is the adherent mode, so that the formation of biofilms by endogenous vaginal lactobacilli is very likely and even desirable. Indeed, the establishment of biofilms of beneficial microbes on human mucosae and epithelia favors their persistence and, possibly, functionality.

Most of the tested CFS were able to reduce *Candida* spp. morphology switching, with bf-CFS recovered from *L. crispatus* strains the most effective. Regarding biofilm formation, different methods of biofilm analysis were employed, since the literature frequently underlined limits of each method, and also in relation to different *Candida* species [23,31]. Crystal violet staining is probably the most common method for biofilm quantification, due to its easiness of execution and low cost. Nevertheless, problems in staining reproducibility have been frequently reported, besides poor selectivity for cells over biofilm extracellular matrix. MTT assay is intended to evaluate only metabolically active cells, giving the chance to quantitate the viable cellular component of the biofilm over the inert matrix.

When we applied crystal violet staining, we demonstrated a marked anti-biofilm activity of CFS recovered from *L. vaginalis*, *L. crispatus*, and *L. plantarum,* especially for bf-CFS, while bf-CFS from *L. gasseri* strains showed the lowest inhibiting activity. The best anti-biofilm profile was also shown for CFS recovered from *L. crispatus* strains when MTT assay was employed, demonstrating a high accordance between the two methods. In addition, CFS recovered from *L. crispatus* strains proved to be significantly more effective than those from *L. gasseri*.

Notably, most *Lactobacillus* CFS determined an increase in CV staining of *C. tropicalis* SO24 and *C. krusei* SO26 biofilms, indicating a stimulating effect. When the same samples were analyzed by MTT assay, such a stimulating effect was not observed. On the contrary, an inhibitory effect was shown. Such inconsistency of the results obtained by CV staining and MTT assay can be explained considering that crystal violet dye also binds to the extracellular matrix, and thus quantifies biofilms as the complex of biomass and matrix, while MTT assay only responds to active cells. It can be speculated that treatment of *C. tropicalis* SO24 and *C. krusei* SO26 biofilms with *Lactobacillus* CFS reduced the viable cellular component of *Candida* biofilms, albeit increasing the accumulation of extracellular material.

*Candida* biofilms were also visualized by confocal microscopy by using dual fluorescent staining, in order to gain information on structure and architecture. *Candida* isolates employed in the present study are able to form biofilms in vitro, although only *C. albicans* isolates and *C. parapsilosis* SO27 built thick structured biofilms, while other isolates showed thinner adherent layers. Overall, the treatment with *Lactobacillus* CFS impedes the formation of such biofilm structures, allowing only the adhesion of small cellular clusters made up of both viable and dead cells. By the confocal microscopy technique, in our experimental conditions, no appreciable differences in biofilm inhibition level could be observed, but only qualitative profiles.

Anti-*Candida* activity of *Lactobacillus* supernatants has been frequently correlated to the production of organic acids, especially lactic acid, and resulting vaginal pH lowering [32,33]. Nevertheless, other mechanisms have been proposed, such as the release of bacteriocin-like substances and biosurfactants. In the present study, a possible antagonistic effect due to the acidic feature of CFS was suppressed by the employment of buffered RPMI medium, as also suggested by EUCAST guidelines for antifungal tests [22], although an enhancement of the anti-*Candida* effect in acidic conditions cannot be excluded. The observed inhibitory activity of *Lactobacillus* CFS towards *Candida* dimorphic switching and biofilms should thus be ascribed to other compounds produced by bacterial metabolism.

Here, we demonstrated that antagonist activity towards *Candida* dimorphic switching and biofilm formation was higher for CFS from *L. crispatus* strains than for those recovered by *L. gasseri* strains, and bf-CFS showed the best inhibition percentages. Such a profile of anti-*Candida* activity resembles the results obtained on *Candida* planktonic growth, which identified bf-CFS from *L. crispatus* BC1–BC7 as the most active, along with bf-CFS from *L. plantarum* BC18 and BC19 [20]. In the same study, the composition of *Lactobacillus* bf-CFS and pk-CFS has been analyzed by ^1^H-NMR. The above-mentioned bf-CFS were characterized by lower amounts of glucose and fructose and higher amounts of galactose with respect to non-active CFS. We can thus speculate that carbohydrate metabolism and preferential consumption of glucose/fructose can be involved in the functional activity. Besides metabolic features of individual *Lactobacillus* species, other mechanisms supporting the observed anti-*Candida* activity could be involved, such as the release of bacteriocin-like molecules or biosurfactants which can prevent the adhesion of *Candida* cells to the surface and, thus, biofilm formation. In this respect, there is poor information on the presence and amount of bacteriocins or biosurfactants in *Lactobacillus* supernatants and further studies based on untargeted proteomic or metabolomic analysis are needed. In addition, *Lactobacillus* supernatants could impair *Candida* virulence factors by modulating the transcription of genes involved in adhesion and biofilm formation, although effector molecules responsible for this function have not been identified yet.

These findings, although obtained in in vitro tests, underline the potential of *L. crispatus* strains as beneficial microbes and strengthen the identification of *L. crispatus* species as a hallmark of vaginal eubiosis [34,35]. Therefore, strains belonging to this species should be further characterized for their safety in cellular models and in in vivo studies, in the perspective of employing probiotic strains to prevent VVC and RVVC or to restore vaginal homeostasis. Furthermore, the fact that *L. crispatus* strains showed enhanced anti-*Candida* activity when grown in the biofilm form confirmed that the formation of biofilms by *L. crispatus* cells is highly desirable in the vaginal niche, in order to improve not only persistence, but also functional activity. In the light of possible applications, these results make this species a good candidate in the development of new-generation probiotics, so called “fourth-generation probiotics”, which are based on strains endowed with good aptitude to form biofilms and suitable to encapsulation [36].

## Figures and Tables

**Table 1:** Inhibition of *Candida* morphology switching by lactobacilli pk-CFS and bf-CFS. +, inhibition of germ tube, pseudohyphae, or hyphae formation, evaluated by microscopic observation of at least three microscopic fields; -, absence of inhibition (i.e., formation of germ tubes, pseudohyphae, or hyphae comparable to the corresponding control sample).

		*C. albicans* SO1	*C. albicans* SO2	*C. lusitaniae* SO22	*C. tropicalis* SO24	*C. krusei* SO26	*C. parapsilosis* SO27
*L. crispatus*	BC1pk	+	+	+	-	+	-
	BC1bf	+	+	+	-	+	+
	BC3pk	+	-	-	-	-	-
	BC3bf	+	+	+	-	-	+
	BC4pk	+	+	-	-	+	-
	BC4bf	+	+	+	-	+	+
	BC5pk	+	+	+	-	+	-
	BC5bf	+	+	+	-	+	+
	BC6pk	+	+	+	-	+	-
	BC6bf	+	+	+	-	+	+
	BC7pk	+	+	-	-	+	-
	BC7bf	+	+	-	-	+	+
	BC8pk	+	-	-	-	-	-
	BC8bf	+	+	+	-	-	+
*L. gasseri*	BC9pk	+	+	+	-	-	+
	BC9bf	-	-	-	-	-	-
	BC1pk	+	+	+	-	+	-
	BC1bf	+	+	+	-	+	-
	BC11pk	+	+	-	-	-	-
	BC11bf	+	+	-	-	+	-
	BC12pk	+	+	-	-	-	-
	BC12bf	+	+	-	-	-	-
	BC13pk	+	-	+	-	-	-
	BC13bf	+	-	+	-	-	-
	BC14pk	+	+	-	-	+	-
	BC14bf	+	+	-	-	-	-
*L. vaginalis*	BC16pk	-	-	-	-	-	-
	BC16bf	-	-	-	-	-	-
	BC17pk	-	+	-	-	+	-
	BC17bf	-	+	-	-	-	-
*L. plantarum*	BC18pk	+	+	-	-	+	-
	BC18bf	+	+	-	-	+	+
	BC19pk	-	+	-	-	+	-
	BC19bf	+	+	-	-	-	+

## Data Availability

Not applicable.

## Supplementary Materials

The following supporting information can be downloaded at: https://www.mdpi.com/article/10.3390/microorganisms10102091/s1, Figure S1: MTT incubation time optimization; Figure S2: Evaluation of *Candida* cell morphology switching; Figure S3: Confocal microscopy analysis of *Candida* biofilm.

## References

[1] Nobile C.J., Johnson A.D. (2015). Candida albicans Biofilms and Human Disease. Annu. Rev. Microbiol..

[2] Willems H.M.E., Ahmed S.S., Liu J., Xu Z., Peters B.M. (2020). Vulvovaginal Candidiasis: A Current Understanding and Burning Questions. J. Fungi.

[3] Rodríguez-Cerdeira C., Gregorio M.C., Molares-Vila A., López-Barcenas A., Fabbrocini G., Bardhi B., Sinani A., Sánchez-Blanco E., Arenas-Guzmán R., Hernandez-Castro R. (2019). Biofilms and vulvovaginal candidiasis. Colloids Surf. B. Biointerfaces.

[4] Blostein F., Levin-Sparenberg E., Wagner J., Foxman B. (2017). Recurrent vulvovaginal candidiasis. Ann. Epidemiol..

[5] Ardizzoni A., Wheeler R.T., Pericolini E. (2021). It Takes Two to Tango: How a Dysregulation of the Innate Immunity, Coupled with Candida Virulence, Triggers VVC Onset. Front. Microbiol..

[6] Atiencia-Carrera M.B., Cabezas-Mera F.S., Tejera E., Machado A. (2022). Prevalence of biofilms in *Candida* spp. bloodstream infections: A meta-analysis. PLoS ONE.

[7] Tsui C., Kong E.F., Jabra-Rizk M.A. (2016). Pathogenesis of Candida albicans biofilm. Pathog. Dis..

[8] Kean R., Delaney C., Rajendran R., Sherry L., Metcalfe R., Thomas R., McLean W., Williams C., Ramage G. (2018). Gaining insights from Candida biofilm heterogeneity: One size does not fit all. J. Fungi.

[9] Tulasidas S., Rao P., Bhat S., Manipura R. (2018). A study on biofilm production and antifungal drug resistance among Candida species from vulvovaginal and bloodstream infections. Infect. Drug Resist..

[10] Sobel J.D. (2015). Editorial Commentary: Vaginal Biofilm: Much Ado about Nothing, or a New Therapeutic Challenge?. Clin. Infect. Dis..

[11] Jacobsen I.D., Wilson D., Wächtler B., Brunke S., Naglik J.R., Hube B. (2012). Candida albicans dimorphism as a therapeutic target. Expert Rev. Anti. Infect. Ther..

[12] Borges S., Silva J., Teixeira P. (2014). The role of lactobacilli and probiotics in maintaining vaginal health. Arch. Gynecol. Obstet..

[13] Pacha-Herrera D., Erazo-Garcia M.P., Cueva D.F., Orellana M., Borja-Serrano P., Arboleda C., Tejera E., Machado A. (2022). Clustering Analysis of the Multi-Microbial Consortium by Lactobacillus Species Against Vaginal Dysbiosis Among Ecuadorian Women. Front. Cell. Infect. Microbiol..

[14] Petrova M.I., Lievens E., Malik S., Imholz N., Lebeer S. (2015). Lactobacillus species as biomarkers and agents that can promote various aspects of vaginal health. Front. Physiol..

[15] Parolin C., Marangoni A., Laghi L., Foschi C., Palomino R.A.Ñ., Calonghi N., Cevenini R., Vitali B. (2015). Isolation of vaginal lactobacilli and characterization of anti-candida activity. PLoS ONE.

[16] Parolin C., Abruzzo A., Giordani B., Oliver J.C., Marangoni A., Luppi B., Vitali B. (2021). Anti-candida activity of hyaluronic acid combined with lactobacillus crispatus lyophilised supernatant: A new antifungal strategy. Antibiotics.

[17] De Gregorio P.R., Parolin C., Abruzzo A., Luppi B., Protti M., Mercolini L., Silva J.A., Giordani B., Marangoni A., Nader-Macías M.E.F. (2020). Biosurfactant from vaginal Lactobacillus crispatus BC1 as a promising agent to interfere with Candida adhesion. Microb. Cell Fact..

[18] Abruzzo A., Giordani B., Parolin C., Vitali B., Protti M., Mercolini L., Cappelletti M., Fedi S., Bigucci F., Cerchiara T. (2018). Novel mixed vesicles containing lactobacilli biosurfactant for vaginal delivery of an anti- Candida agent. Eur. J. Pharm. Sci..

[19] Abruzzo A., Giordani B., Parolin C., De Gregorio P.R., Foschi C., Cerchiara T., Bigucci F., Vitali B., Luppi B. (2021). Lactobacillus crispatus BC1 biosurfactant delivered by hyalurosomes: An advanced strategy to counteract candida biofilm. Antibiotics.

[20] Parolin C., Croatti V., Laghi L., Giordani B., Tondi M.R., De Gregorio P.R., Foschi C., Vitali B. (2021). Lactobacillus Biofilms Influence Anti-Candida Activity. Front. Microbiol..

[21] Oliver J.C., Laghi L., Parolin C., Foschi C., Marangoni A., Liberatore A., Dias A.L.T., Cricca M., Vitali B. (2020). Metabolic profiling of Candida clinical isolates of different species and infection sources. Sci. Rep..

[22] EUCAST Committee Method for the determination of broth dilution minimum inhibitory concentrations of antifungal agents for yeasts. EUCAST Defin. Doc. E.DEF 7.3.2 **2020**.

[23] Rutering J., Ilmer M., Recio A., Coleman M., Vykoukal J., Alt E., Orleans N. (2016). Quantitative and Qualitative Assessment Methods for Biofilm Growth: A Mini-review Christina. Nat. Rev. Drug Discov..

[24] Aballéa S., Guelfucci F., Wagner J., Khemiri A., Dietz J.P., Sobel J., Toumi M. (2013). Subjective health status and health-related quality of life among women with Recurrent Vulvovaginal Candidosis (RVVC) in Europe and the USA. Health Qual. Life Outcomes.

[25] Han C., Li H., Han L., Wang C., Yan Y., Qi W., Fan A., Wang Y., Xue F. (2019). Aerobic vaginitis in late pregnancy and outcomes of pregnancy. Eur. J. Clin. Microbiol. Infect. Dis..

[26] Dover S.E., Aroutcheva A.A., Faro S., Chikindas M.L. (2008). Natural antimicrobials and their role in vaginal health: A short review. Int. J. Probiotics Prebiotics.

[27] Fanning S., Mitchell A.P. (2012). Fungal biofilms. PLoS Pathog..

[28] Mitchell K.F., Zarnowski R., Andes D.R. (2016). Fungal Super Glue: The Biofilm Matrix and Its Composition, Assembly, and Functions. PLoS Pathog..

[29] Desai J.V. (2018). Candida albicans hyphae: From growth initiation to invasion. J. Fungi.

[30] Liu L., Guo S., Chen X., Yang S., Deng X., Tu M., Tao Y., Xiang W., Rao Y. (2021). Metabolic profiles of Lactobacillus paraplantarum in biofilm and planktonic states and investigation of its intestinal modulation and immunoregulation in dogs. Food Funct..

[31] Lohsea M., Megha G., Arevalo A.V., Fishburn A., Johnson A., Nobile C. (2017). Assessment and Optimizations of Candida albicans In Vitro Biofilm Assays. Antimicrob. Agents Chemother..

[32] O’Hanlon D.E., Moench T.R., Cone R.A. (2013). Vaginal pH and microbicidal lactic acid when lactobacilli dominate the microbiota. PLoS ONE.

[33] De Gregorio P., Silva J., Marchesi A., Nader-Macías M. (2019). Anti-Candida activity of beneficial vaginal lactobacilli in in vitro assays and in a murine experimental model. FEMS Yeast Res..

[34] Parolin C., Frisco G., Foschi C., Giordani B., Salvo M., Vitali B., Marangoni A., Calonghi N. (2018). Lactobacillus crispatus BC5 interferes with Chlamydia trachomatis infectivity through integrin modulation in cervical cells. Front. Microbiol..

[35] Argentini C., Fontana F., Alessandri G., Lugli G.A., Mancabelli L., Ossiprandi M.C., van Sinderen D., Ventura M., Milani C., Turroni F. (2022). Evaluation of Modulatory Activities of Lactobacillus crispatus Strains in the Context of the Vaginal Microbiota. Microbiol. Spectr..

[36] Salas-Jara M., Ilabaca A., Vega M., García A. (2016). Biofilm Forming Lactobacillus: New Challenges for the Development of Probiotics. Microorganisms.

