# Supplementary material for: Vaginal Lactobacillus Impair Candida Dimorphic Switching and Biofilm Formation
Source: Microorganisms. 2022 Oct 21;10(10):2091. doi: 10.3390/microorganisms10102091 (PMC9609122; doi:10.3390/microorganisms10102091)

microorganisms

MDPI

Supplementary Materials

Article: **Vaginal *Lactobacillus* impair *Candida* dimorphic switching and biofilm formation**

Carola Parolin*, Vanessa Croatti, Barbara Giordani, Beatrice Vitali

Figure S1. MTT incubation time optimization. Starting from agar cultures, *Candida* suspensions were prepared in supplemented RPMI at the final concentrations of 1–5 × 10^7, 1–5 × 10^6 and 1–5 × 10^5 CFU/mL and inoculated in flat-bottomed 96-well plates (0.1 mL per well), added with the same volume of MRS medium. Plates were incubated at 35°C in aerobic conditions for 72 h. *Candida* biofilms were assayed with 0.5% MTT as described in the materials and methods and different incubation times were tested: 0.5, 1, 2, 3, 4 and 7 h. The absorbance measured at 570 nm using a GENios Plate Reader are reported (average of at least three replicates ± SEM). Optimum incubation time was defined for each *Candida* isolate, considering a value of Abs 570nm in the range 0.5-1.5 for 1-5 × 10^6 CFU/mL suspension (which corresponded to the starting inoculum in experiments with CFS). Values over the limit of the instrument are arbitrarily indicated as 3 in the figures. Incubation times of 4 h for *C. albicans*, 3 h for *C. glabrata*, 0.5 h for *C. lusitaniae* and *C. parapsilosis*, and 2 h for *C. tropicalis* and *C. krusei* were selected.

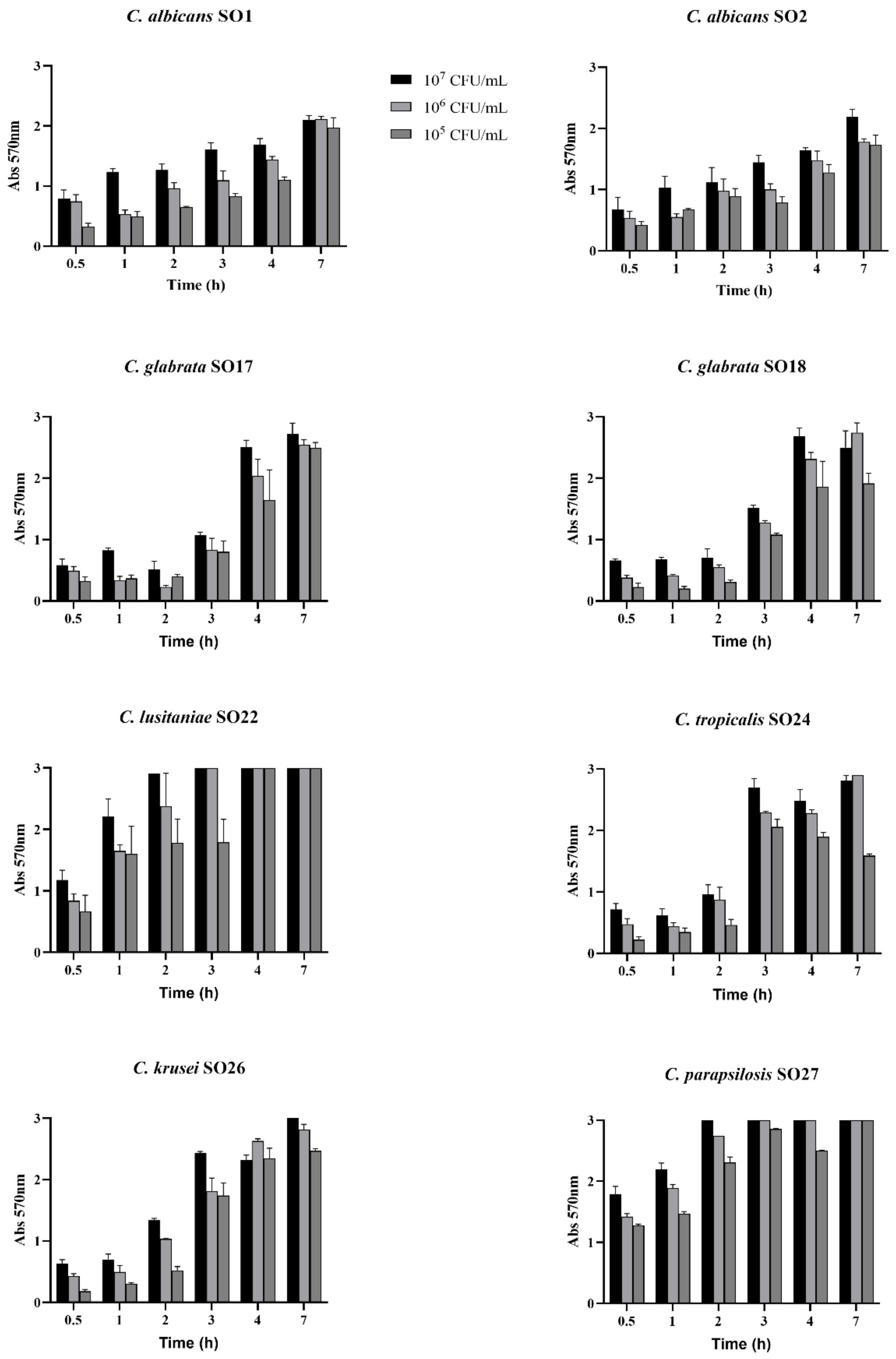
C. albicans SO1
C. albicans SO2
C. glabrata SO17
C. glabrata SO18
C. lusitaniae SO22
C. tropicalis SO24
C. krusei SO26
C. parapsilosis SO27
10^7 CFU/mL
10^6 CFU/mL
10^5 CFU/mL
Abs 570nm
Time (h)
0.5
1
2
3
4
7

Figure S2. Evaluation of Candida cell morphology switching. Images from optical microscope (20X) of *Candida* germ tube/Pseudohyphae/hyphae in presence or not of *L. crispatus* BC3 and *L. gasseri* BC9 pk and bf-CFS. Rows represent different *Candida* strains while columns reported controls and treatments, as indicated above the micrographs.
(**A**) *C. lusitaniae* SO22. (**B**) *C. tropicalis* SO24. (**C**) *C. krusei* SO26. (**D**) *C. parapsilosis* SO27. Scale bar = 50 µm.

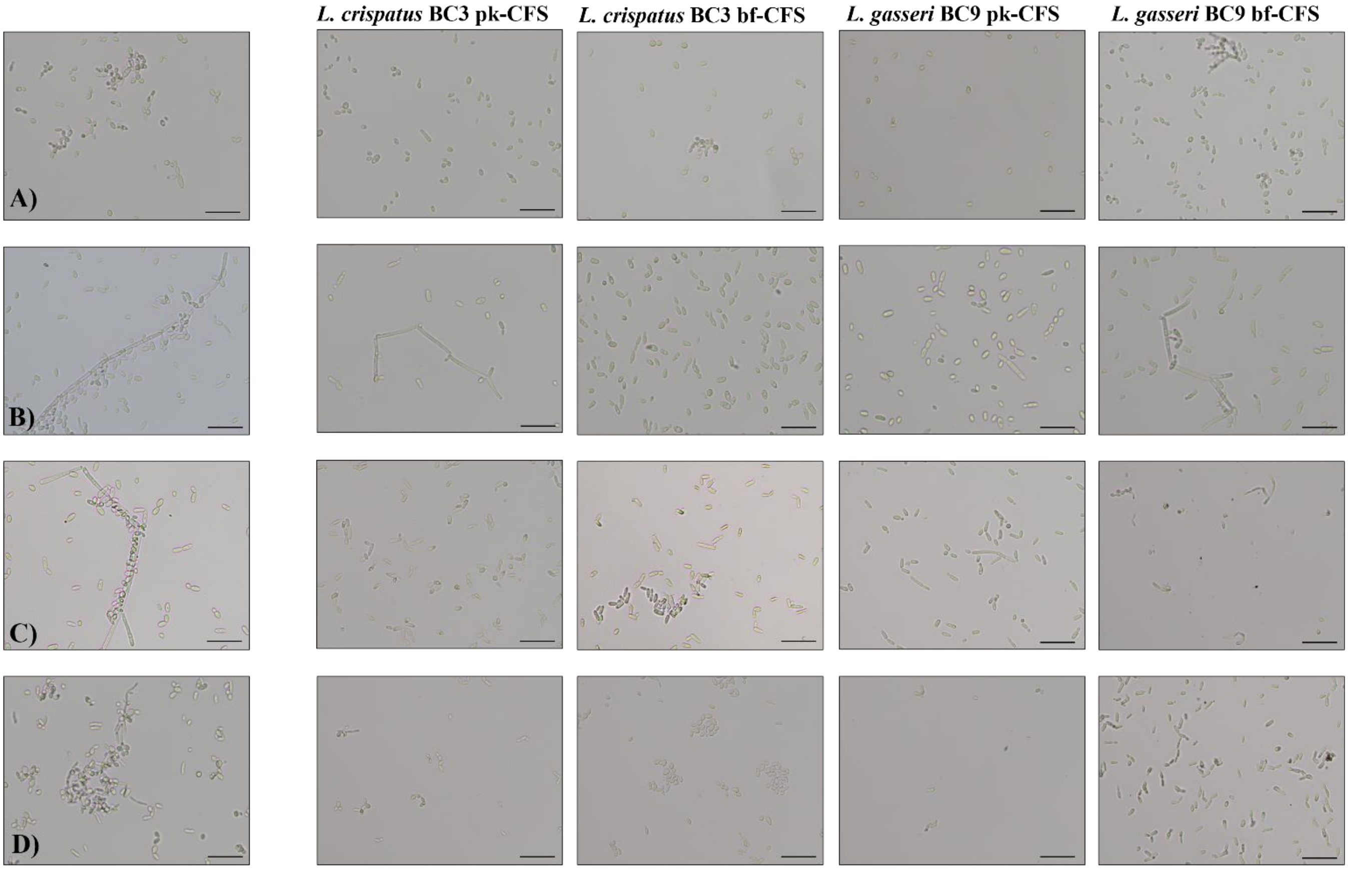

microorganisms

Figure S3. Confocal microscopy analysis of *Candida* biofilm. *Candida* spp. were treated with pk-CFS and bf-CFS from *L. crispatus* BC5 and *L. gasseri* BC12 (second-fifth columns). Biofilms were stained using two fluorescent dyes: thiazole orange (viable cells, green) and Live-or-Dye™ (dead cells, red). (**A**) *C. glabrata* SO18 biofilm. (**B**) *C. parapsilosis* SO27 biofilm. Scale bar = 100 µm.

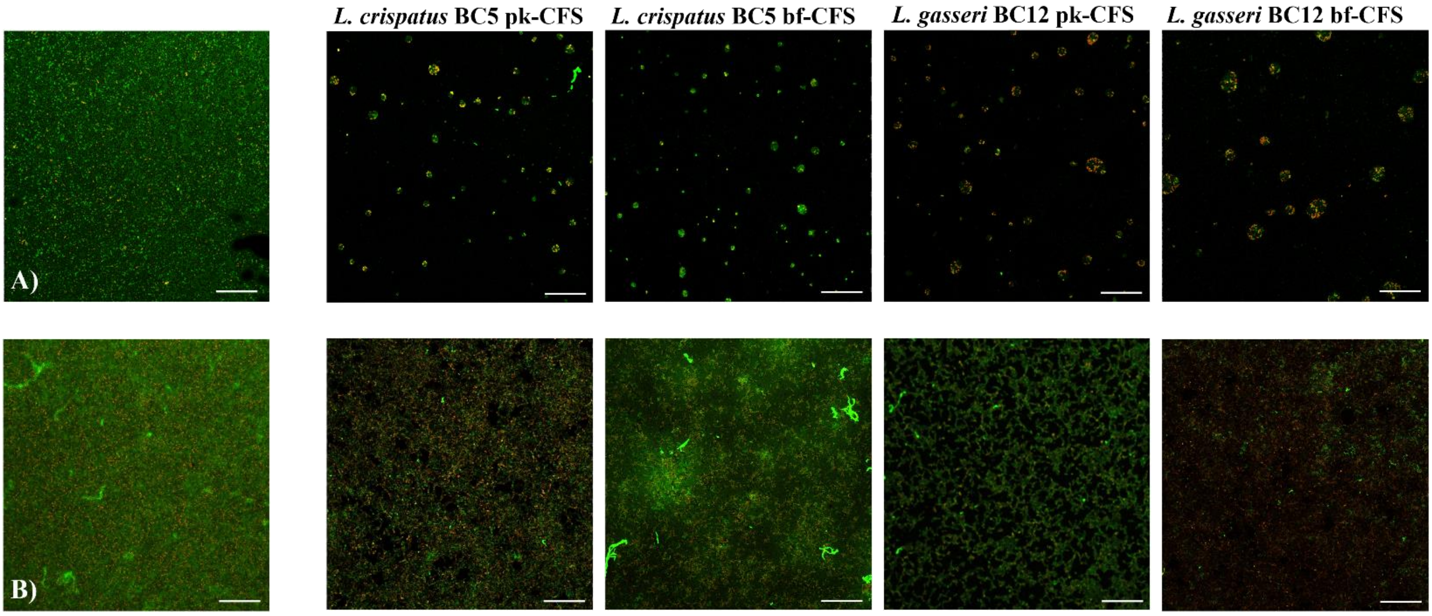

Supplement: Supplementary file 1 [file microorganisms-10-02091-s001.zip › microorganisms-1935716-supplementary.pdf]
